# Association Between Patient-Reported Outcomes and Overall Survival in Patients with Advanced NSCLC Treated with First-Line Cemiplimab-Based Therapy

**DOI:** 10.3390/cancers18060916

**Published:** 2026-03-12

**Authors:** David R. Gandara, Tamta Makharadze, Mahmut Gümüş, Miranda Gogishvili, Ahmet Sezer, Eric Kim, Eric Yan, Frank Seebach, James Harnett, Ruben G. W. Quek

**Affiliations:** 1 Division of Hematology/Oncology, Department of Medicine, UC Davis Comprehensive Cancer Center, 2279 45th Street, Sacramento, CA 95817, USA; 2 LTD High Technology Hospital Medical Center, 118 Pushkini Street, 6000 Batumi, Georgia; 3 Department of Medical Oncology, School of Medicine, Istanbul Medeniyet University, Dumlupınar D100 Karayolu No. 98, 34720 Kadiköy, Istanbul, Turkey; 4 High Technology Medical Centre, University Clinic Ltd., 9 Tsinandali Street, 0144 Tbilisi, Georgia; 5 Department of Medical Oncology, Başkent University, Kazım Karabekir, Gülhatmi Cd. 37/A, 01120 Adana, Adana Province, Turkey; 6 Regeneron Pharmaceuticals, Inc., 777 Old Saw Mill River Road, Tarrytown, NY 10591, USA; 7 Cyan Global Inc., 5965 Village Way STE 105-118, San Diego, CA 92130, USA

**Keywords:** patient-reported outcomes, cemiplimab, non-small-cell lung cancer, immunotherapy

## Abstract

**Background/Objectives**: Research on the association between change from baseline in patient-reported outcomes (PROs) and overall survival (OS) in patients with advanced non-small-cell lung cancer (NSCLC) exists. This study evaluated the association between post-baseline PROs and OS in patients with advanced NSCLC who received first-line cemiplimab-based therapy. **Methods**: We evaluated PRO data from two phase III studies (EMPOWER-Lung 1 [NCT03088540] and EMPOWER-Lung 3 [NCT03409614]) using a Cox proportional hazards model. Twelve pre-specified PRO scales from the European Organisation for Research and Treatment of Cancer Quality of Life Questionnaire Core 30 and Quality of Life Questionnaire Lung Cancer 13 module were evaluated. Landmark analyses were conducted at 3, 6, 9, and 12 months. Time-dependent analyses using change from baseline PROs as a time-dependent covariate were conducted to evaluate the association of post-baseline PRO improvement with OS. **Results**: At the 3-month landmark, we observed a 56% reduction in the risk of death (HR = 0.44; 95% CI: 0.32–0.62; nominal *p* < 0.0001) among stable/improved vs. worsened/unobserved PROs for global health status (GHS)/quality of life (QoL). Results at the 6-, 9-, and 12-month landmarks of GHS/QoL were consistent with those at the 3-month landmark. Time-dependent analyses showed that every 10-point improvement in GHS/QoL was associated with a 31% reduction in the risk of death (HR = 0.69; 95% CI: 0.64–0.75; nominal *p* < 0.0001). **Conclusions**: In patients with advanced NSCLC who received first-line cemiplimab-based therapy, improvements in post-baseline PROs are associated with improved OS. These results may inform endpoint selection and interpretation of future clinical trials.

## Simple Summary

Patient-reported outcomes (PROs) are emerging as an important endpoint in clinical trials, facilitating interpretation and clinical application. Previous studies have examined the association between baseline PROs and overall survival, providing evidence for the potential prognostic value. However, limited research has investigated these associations in the context of immune checkpoint inhibitor treatment for advanced non-small-cell lung cancer, particularly regarding the relationship between changes from baseline in PROs and overall survival. To explore this relationship and its potential clinical relevance, the current study evaluated the association between sequential post-baseline PROs and overall survival in patients with advanced non-small-cell lung cancer who received first-line cemiplimab-based therapy.

## 1. Introduction

Lung cancer remains the most commonly diagnosed cancer worldwide, and the leading cause of cancer-related mortality, with substantial impact on both survival and patient quality of life. While advances in immunotherapy have improved outcomes, patients often experience a complex balance between treatment benefit and symptom burden [1,2]. Many studies that evaluate novel therapies for the treatment of cancer have traditionally focused on efficacy outcomes such as tumor response, progression-free survival, and overall survival (OS). However, while these endpoints remain the standard, they may not fully capture the patient experience. Patient-reported outcomes (PROs) are also important sources of data, with their integration into routine clinical practice being well established. Of particular interest is their potential use as prognostic indicators of survival [3]. A number of papers on baseline PRO associations with OS outcomes in cancer have previously been published [3,4,5]. More recently, a systematic review and meta-analysis evaluated the association between baseline PRO measures and OS, and found that PROs offer independent prognostic information beyond traditional clinical outcomes for cancer survival [6].

While associations between OS and baseline PROs have been explored in the broader oncology literature, there have been limited publications of such associations regarding immune checkpoint inhibitors in advanced non-small-cell lung cancer (NSCLC) [7]. In addition, research on the association between change from baseline PRO data at post-baseline visits and OS is limited [5,7]. The available evidence points towards a prognostic utility of PROs that warrants further research [4,7,8].

Cemiplimab, a monoclonal antibody that targets programmed cell death-1 (PD-1), has been approved in the United States as a first-line treatment of adult patients with advanced NSCLC. This includes patients with locally advanced disease and who are not candidates for surgical resection or definitive chemoradiotherapy, as well as those with metastatic NSCLC with no *EGFR*, *ALK*, or *ROS1* aberrations. Cemiplimab is used as a single agent in tumors expressing programmed cell death-ligand 1 (PD-L1) at levels of 50% or higher (as determined by an FDA-approved test), and in combination with platinum-based chemotherapy regardless of the PD-L1 expression level [9]. This approval was based on the results from two international pivotal phase III trials: EMPOWER-Lung 1 (NCT03088540) in patients with advanced NSCLC and PD-L1 50% or higher who received cemiplimab monotherapy; and EMPOWER-Lung 3 (Part 2; NCT03409614) in patients with advanced NSCLC who received cemiplimab in combination with chemotherapy (Table 1; Supplementary Figure S1) [9,10,11]. A recent study demonstrated that baseline PROs can significantly predict survival in patients with advanced NSCLC treated with cemiplimab [12]. These PRO data were collected using the cancer-specific European Organisation for Research and Treatment of Cancer (EORTC) Quality of Life Questionnaire Core 30 (QLQ-C30) and the disease-specific EORTC QLQ Lung Cancer 13 (QLQ-LC13) modules, the most commonly used PRO instruments in clinical trials of immune checkpoint inhibitors in advanced NSCLC [13].

In a previous study, baseline PROs were shown to have significant prognostic value for survival in patients with advanced NSCLC treated with cemiplimab-based therapy. Several PRO domains, including dyspnea and physical functioning, were significantly associated with OS and demonstrated greater prognostic value than physician-reported Eastern Cooperative Oncology Group performance status [12], highlighting the importance of patient-reported measures as independent predictors of clinical outcomes at treatment initiation.

However, by focusing on baseline PROs, the study did not capture how PROs may change over time. Evaluating longitudinal changes may provide additional clinically relevant prognostic information. The present study aims to build on previous findings and address an important gap in the literature by determining whether improvement or deterioration in PROs during treatment is associated with OS outcomes beyond baseline status.

The primary objective of this study was to evaluate the association between OS and changes from baseline in PROs among patients with advanced NSCLC treated with first-line cemiplimab-based therapy. The study focused on a set of pre-specified PRO scales from the EORTC QLQ-C30 and QLQ-LC13 that were most clinically relevant in patients with advanced NSCLC.

## 2. Materials and Methods

PRO data were collected using the EORTC QLQ-C30 and QLQ-LC13 questionnaires. These data were sourced from the cemiplimab monotherapy arm in EMPOWER-Lung 1, which included 283 patients, and the cemiplimab combined with chemotherapy arm in EMPOWER-Lung 3, comprising 312 patients. The analysis focused on a set of 12 pre-specified PRO scales that were considered most relevant to patients diagnosed with advanced NSCLC (Table 2). From the QLQ-C30, the following domains were evaluated: global health status (GHS)/quality of life (QoL); functioning scales, including physical functioning and role functioning; and symptom scales/items comprising fatigue, pain, dyspnea, and appetite loss. From the QLQ-LC13 module, lung cancer–specific symptom scales were analyzed, including cough, dyspnea, and site-specific pain. Site-specific pain was further examined through its localization: chest pain; pain in the arm or shoulder; and pain in other parts of the body. All PROs were assessed among patients randomized to a study treatment; the longitudinal analysis included those with a baseline and at least one post-baseline assessment.

PROs were assessed at several different timepoints to capture longitudinal changes over the course of treatment: initially at baseline (Day 1), during each treatment cycle for the first six doses, then subsequently every three cycles, and finally at the end of the treatment regimen. No windowing conventions were applied for the analysis of the PRO data, and patient data were not excluded from analyses owing to the patient’s failure to comply with the visit schedule. Cox proportional hazards models were used to assess the relationship between PROs and OS. The models were stratified by treatment type, tumor histology, and PD-L1 expression level.

Landmark analyses were conducted at several clinically relevant timepoints: 3 months, 6 months, 9 months, and 12 months. Timepoints were based in part on progression-free survival results of the EMPOWER-Lung trials. At each prespecified landmark, PROs were classified according to the status as stable or improved vs. worsened or unobserved. This classification was based on a 10-point threshold [8,14], which represents an established minimal clinically important difference in the EORTC QLQ-C30 and QLQ-LC13 PRO scales used (Table 3). In addition to the landmark analyses, time-dependent analyses using change from baseline PROs as a time-dependent covariate were also conducted to provide a more dynamic assessment of the association of post-baseline PRO improvement with OS. Patients who did not experience clinically meaningful deterioration were censored at the date of the last available assessment (the date of the last non-missing value). Patients with no baseline assessment were censored at the date of randomization. Death or progression will not be considered deterioration events in the primary analysis.

## 3. Results

**Figure 1:**
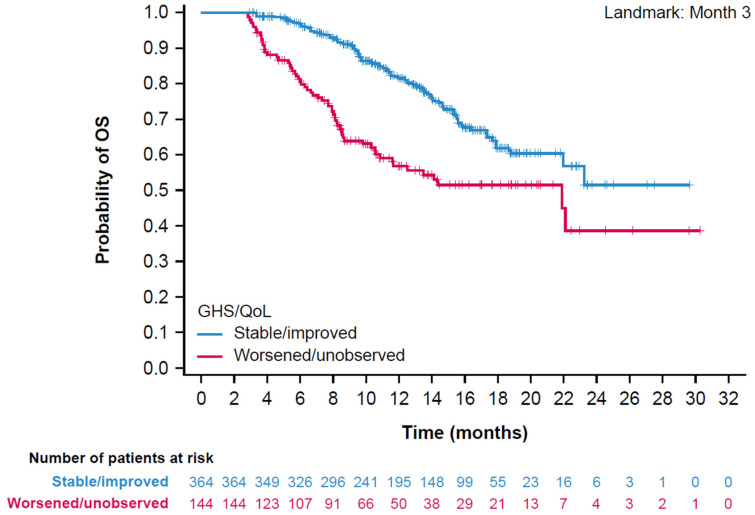
Kaplan–Meier plot of OS by stable/improved vs. worsened/unobserved PROs in GHS/QoL at 3 months. GHS, global health status; QoL, quality of life; OS, overall survival; PRO, patient-reported outcome.

At the prespecified 3-month landmark analysis, patients with stable or improved PROs experienced a 56% reduction in risk of death compared with patients with worsened or unobserved PROs GHS/QoL, with a hazard ratio (HR) of 0.44 (95% confidence interval [CI]: 0.32–0.62; nominal *p* < 0.0001). A Kaplan–Meier plot shows clear separation of OS between stable or improved and worsened or unobserved PROs for GHS/QoL at 3 months (Figure 1). Results at the 6-month, 9-month, and 12-month landmarks of GHS/QoL were consistent with those at the 3-month landmark (Table 4; Supplementary Figure S2). At the prespecified 6-month landmark analysis, improvement in GHS/QoL was significantly associated with improved OS, with an HR of 0.29 (95% CI: 0.20–0.42; *p* < 0.0001). This corresponds to a 71% reduction in the risk of death among patients who experienced improvement in GHS/QoL compared with worsened or unobserved PROs. This association persisted at the 9-month landmark with an HR of 0.14 (95% CI: 0.07–0.27; *p* < 0.0001), representing an 86% reduction in risk of death, and at the 12-month landmark a 90% reduction in the risk of death (HR 0.10, 95% CI: 0.04–0.26; *p* < 0.0001).

The PRO completion rate at each of the 3-, 6-, 9-, and 12-month landmark time points was ≥94%. A decreased risk of death among patients with stable or improved PROs, compared to those with worsened or unobserved PROs, was also observed for additional PRO scales at the 3-month landmark assessment including physical functioning (HR 0.45, 95% CI: 0.32–0.63; *p* < 0.0001) and pain (HR 0.39, 95% CI: 0.28–0.55; *p* < 0.0001) (Table 5). Scales from the EORTC-QLQ-LC13 also showed a reduction in the risk of death, including LC-dyspnea (HR 0.61, 95% CI: 0.44–0.85; *p* = 0.0037), LC-coughing (HR 0.45, 95% CI: 0.32–0.65; *p* < 0.0001), and LC-pain in chest (HR 0.42, 95% CI: 0.29–0.59; *p* < 0.0001). In alignment with the results observed for GHS/QoL, the OS HRs also consistently and significantly (with nominal *p* < 0.05) favored the group with stable or improved PROs compared with worsened or unobserved outcomes across all other pre-specified scales of interest at the subsequent landmark time points of 6, 9, and 12 months (Supplementary Tables S1–S3). For physical functioning, the landmark analysis at 6 months showed a HR of 0.26 (95% CI: 0.18–0.38; *p* < 0.0001) for patients with stable or improved PROs compared with those with worsened or unobserved PROs. Similarly, at 9 months and 12 months, an 85% and 84% reduction in the risk of death was observed among stable or improved outcomes compared with worsened or unobserved PROs, respectively. For pain, the landmark analysis at 6 months showed an HR of 0.23 (95% CI: 0.16–0.35; *p* < 0.0001) for patients with stable or improved PROs compared with those with worsened or unobserved PROs. Similarly, at 9 months and 12 months, an 83% and 88% reduction in the risk of death was observed among stable or improved compared with worsened or unobserved PROs, respectively.

Time-dependent analyses showed that for each 10-point improvement in the GHS/QoL there was an associated 31% decrease in the risk of death, with an HR of 0.69 (95% CI: 0.64–0.75), which was statistically significant (nominal *p* < 0.0001) (Table 6). Similarly, improvements in functional domains were strongly associated with OS. A 10-point increase in physical functioning was associated with a 26% reduction in mortality risk (HR 0.74, 95% CI: 0.69–0.80; *p* < 0.0001), while role functioning showed similar results. Symptom scales also demonstrated significant associations with OS. Improvements in pain corresponded to a 20% reduction in risk (HR 0.80, 95% CI 0.75–0.85; *p* < 0.0001). Consistent findings were observed for lung cancer–specific symptoms assessed by the QLQ-LC13 module. A 10-point improvement in LC-dyspnea was associated with a 17% reduction in mortality risk (HR 0.83, 95% CI: 0.77–0.88; *p* < 0.0001), and improvements in LC-coughing were associated with a 16% reduction (HR 0.84, 95% CI: 0.79–0.89; *p* < 0.0001). Improvements in several types of pain, including chest pain (HR 0.83, 95% CI 0.78–0.89; *p* < 0.0001), were significantly associated with longer OS.

## 4. Discussion

PROs have become an increasingly important aspect of lung cancer research and routine clinical practice, providing direct insights into patients’ experiences and perceptions of their disease and treatment journey. While mainly used to assess disease-related QoL and treatment-associated toxicities, emerging research suggests that PROs may also provide significant prognostic indicators for key clinical outcomes that are complementary to physician assessments [15]. PROs for first-line cemiplimab as monotherapy or in combination with chemotherapy in phase III studies showed overall improvement in symptoms and delayed time to definitive clinically meaningful deterioration in cancer-related and lung cancer–specific symptoms and functions [16,17]. Prior research has also shown that baseline PROs were prognostic for survival outcomes in patients with advanced NSCLC who initiated first-line cemiplimab-based therapy [12,18]. Additionally, a composite PRO and risk prediction model for OS in advanced NSCLC treated with first-line cemiplimab-based therapy has shown that using baseline composite PROs of one functioning scale and one symptom scale can evaluate OS risk better than single PRO scales [19]. To our knowledge, this current study is the first to evaluate the prognostic correlation between OS and post-baseline changes in PROs using landmark and time-dependent analyses in patients with advanced NSCLC receiving first-line checkpoint immunotherapy.

The value of incorporating PRO data into the investigation of treatments in clinical trials is increasingly recognized by regulatory bodies as well as researchers and clinicians. A group of researchers has proposed a core set of PROs that are key indicators of treatment effect on health-related quality of life to optimize PRO assessment strategy [15]. The US Food and Drug Administration also recently published a collection of recommended core PROs for cancer clinical trials, including physical functioning, role functioning, and disease-related symptoms [20]. Following this guidance, in our study pre-specification of post-baseline changes in GHS/QoL, physical functioning, role functioning, and NSCLC disease-related symptoms from the EORTC QLQ-C30 and EORTC QLQ-LC13 were investigated for association with OS.

Using landmark analyses, our study showed that, in patients with advanced NSCLC who received first-line cemiplimab-based therapy, improvements in post-baseline PROs (measured as change from baseline at 3, 6, and 12 months across all 12 PRO scales most relevant to advanced NSCLC) were strongly associated with improved OS. These statistically significant associations between post-baseline improvement of PROs and OS were also validated by the time-dependent analyses using a 10-point threshold as the minimum clinically important difference.

The OS prognostic significance of GHS/QoL observed in our analysis aligns with a prior publication that identified GHS/QoL as the most frequent predictor of survival among PROs in multiple tumor types [21]. The underlying mechanisms driving this association are likely multifaceted. Patients’ experiences and changes in their GHS/QoL throughout their treatment journey of cemiplimab may capture pathophysiological changes that complement clinical detection and potentially reflect underlying tumor biology, disease burden, or systemic responses to cancer. This hypothesis was reinforced by our research indicating that, for patients with advanced NSCLC treated with cemiplimab, improvements in post-baseline GHS/QoL were significantly associated with improved OS.

Our analysis also revealed that post-baseline improvements in physical functioning and role functioning were associated with improved OS. Patient-reported physical functioning may serve as an important predictor of lung cancer burden and progression, potentially detecting subtle deterioration that is not apparently measured by conventional clinical assessments [5,22]. Even minor worsening in physical capability might indicate cancer progression before becoming apparent through standard imaging or laboratory measures [23,24]. In addition to physical functioning, role functioning also encompasses cognitive and emotional dimensions that are required to maintain work and social responsibilities [25]. Our findings reinforce the importance of comprehensive functional assessment in advanced NSCLC prognostication.

Patient-reported symptom burden and functional status may capture aspects of lung cancer that are not completely understood by clinician assessments alone. Multiple studies have demonstrated that baseline QoL can be a prognostic factor for survival in NSCLC. One study demonstrated that simple single items (clinically deficient score) independently predicted OS in populations with advanced NSCLC, and this association persisted even after controlling for performance status and disease stage [26]. Similarly, in a prospective observational study, higher pretreatment health-related QoL scores were predictive of longer OS, while declining values were associated with worse treatment outcomes [27]. Another study exploring changes in QoL and survival of patients with NSCLC demonstrated that certain symptoms, such as baseline constipation and changes in fatigue over time, were predictive of OS [28]. Notably, the concept of time to deterioration in health-related QoL has been shown to behave similarly to traditional time-to-event endpoints, with health-related QoL decline paralleling disease progression and mortality [29,30]. Similar results have been reported in other cancer types. Analyses from CheckMate 214 among patients with advanced renal cell carcinoma demonstrated that stable or improved health-related QoL had a reduced risk of death compared with worsened or unobserved health-related QoL, regardless of treatment (nivolumab + ipilimumab vs. sunitinib), reinforcing PROs as prognostic indicators of survival [8]. A systematic review and meta-analysis showed that treatment with PD-1/PD-L1 inhibitors not only prolongs OS in patients with NSCLC but also meaningfully improves QoL compared to conventional chemotherapy [2]. Although the instruments differed (QLQ-C30 and EQ-5D-3L), the study demonstrated that PD-1/PD-L1 inhibitors delay clinically significant deterioration in QoL, particularly in domains such as physical function, role function, and pain [2]. Interestingly, a small prospective study using both the EORTC QLQ-C30 and EORTC QLQ-LC-13 instruments found that, even among patients with lung cancer treated with immunotherapy and experiencing immune-related adverse events, QoL was reported as improved [31]. Taken together, these findings position longitudinal PRO monitoring as a potentially actionable prognostic tool in advanced NSCLC.

Overall, our study demonstrates a strong association between post-baseline improvement in PROs and OS across all pre-specified scales, in both landmark analyses and time-dependent analyses, in patients with advanced NSCLC treated with first-line cemiplimab-based therapy. The prognostic value of PROs observed in our study supports their integration into multidisciplinary advanced NSCLC clinical decision-making frameworks. Prioritization of the post-baseline PROs prespecified in this study could be incorporated into predictive models, potentially leveraging advances in machine learning/artificial intelligence to develop more accurate advanced NSCLC OS prediction tools that combine clinical endpoints, biomarkers, and PROs.

One of the strengths of this study is the large sample size (*n* = 595). This study included the patients treated with first-line cemiplimab-based therapy from two large phase III trials, EMPOWER-Lung 1 and EMPOWER-Lung 3. Another key strength of this study was the use of validated PRO instruments, including the EORTC QLQ-C30 and QLQ-LC13, both clinically relevant to patients with NSCLC.

Although the results of this study provide valuable insight, there are limitations to keep in mind. This analysis only examined data from patients with advanced NSCLC who received first-line cemiplimab-based treatment; therefore, the findings may not be generalizable to patient populations of different cancer types and treatments. The Cox proportional hazards models were stratified by treatment type, tumor histology, and PD-L1 expression level. These study results will not be applicable to situations where there are unbalanced data in other key prognostic variables, such as baseline Eastern Cooperative Oncology Group performance status. Our study was not designed to directly compare the prognostic utility across PROs; future research to compare and evaluate the reasons behind differences in prognostic utility of post-baseline PROs will be useful to inform clinical trial endpoint selection and will further improve the individualization and prioritization of PROs according to advanced NSCLC population characteristics. Larger-scale studies designed to assess the association between change from baseline PROs and OS in other tumor and treatment settings are needed to confirm our results. Future studies should also investigate the comparability of results among subgroups of patients (e.g., monotherapy vs. combination; PD-L1 ≥ 50% vs. <50% levels). PROs collected from patients with advanced NSCLC in a real-world setting would also be valuable to validate these study findings [32].

## 5. Conclusions

In patients with advanced NSCLC treated with first-line cemiplimab-based therapy, improvements in post-baseline PROs are associated with improved OS. The study findings may have important implications for the design and interpretation of future clinical trials, helping to inform endpoint selection and interpretation, as well as providing early indications of the potential clinical benefit of immunotherapies in advanced NSCLC.

## Figures and Tables

**Table 1:** Overview of the EMPOWER-Lung 1 and EMPOWER-Lung 3 clinical trial study designs.

	EMPOWER-Lung 1 [10]		EMPOWER-Lung 3 Part 2 [11]	
**Patient population**	Patients with advanced NSCLC with PD-L1 expression level ≥ 50%		Patients with advanced NSCLC with any PD-L1 expression level	
**Randomization**	1:1		2:1	
**Treatment arm**	Arm A	Arm B	Arm A	Arm B
**Study treatment**	Cemiplimab	Chemotherapy	Cemiplimab + chemotherapy	Placebo + chemotherapy
**Primary endpoints**	OS, PFS		OS	

NSCLC, non-small-cell lung cancer; OS, overall survival; PD-L1, programmed cell death-ligand 1; PFS, progression-free survival.

**Table 2:** Pre-specified PRO scales of interest most relevant to advanced NSCLC.

From the EORTC-QLQ-C30	From the EORTC-QLQ-LC13
GHS/QoL	
**Functioning scales:**	**Lung cancer symptoms:**
Physical functioning	Cough
Role functioning	Dyspnea
**Symptom scales/items:**	**Site-specific pain:**
Fatigue	Pain in chest
Pain	Pain in arm or shoulder
Dyspnea	Pain in other parts
Appetite loss	

EORTC, European Organisation for Research and Treatment of Cancer; GHS, global health status; QLQ-C30, Quality of Life Questionnaire Core 30; QLQ-LC13, Quality of Life Questionnaire Lung Cancer 13; QoL, quality of life.

**Table 3:** Definitions of improved, stable, worsened, and unobserved.

Scale	Change from Baseline	Visit Response ^1^
GHS/QoL and functioning	≥+10	Improved
	≤−10	Worsened
	Otherwise	Stable
Symptom	≥+10	Worsened
	≤−10	Improved
	Otherwise	Stable

^1^ Visit response was classified as unobserved if a patient was still in the study, but no PRO data were observed. GHS, global health status; PRO, patient-reported outcome; QoL, quality of life.

**Table 4:** The 6-, 9-, and 12-month landmark analyses of GHS/QoL.

Landmark Timepoint	HR (95% CI)	*p*-Value
6 months (*n* = 444)	0.29 (0.20–0.42)	<0.0001
9 months (*n* = 321)	0.14 (0.07–0.27)	<0.0001
12 months (*n* = 251)	0.10 (0.04–0.26)	<0.0001

CI, confidence interval; GHS, global health status; HR, hazard ratio; QoL, quality of life.

**Table 5:** OS HRs of stable or improved PROs vs. worsened or unobserved PROs: Landmark analysis at 3 months (*n* = 508).

Variable	HR (95% CI)	*p*-Value
GHS/QoL	0.44 (0.32–0.62)	<0.0001
Physical functioning	0.45 (0.32–0.63)	<0.0001
Role functioning	0.57 (0.41–0.79)	0.0009
Fatigue	0.51 (0.37–0.71)	<0.0001
Pain	0.39 (0.28–0.55)	<0.0001
Dyspnea	0.38 (0.27–0.54)	<0.0001
Appetite loss	0.40 (0.29–0.56)	<0.0001
LC-Dyspnea	0.61 (0.44–0.85)	0.0037
LC-Coughing	0.45 (0.32–0.65)	<0.0001
LC-Pain in arm or shoulder	0.44 (0.31–0.62)	<0.0001
LC-Pain in chest	0.42 (0.29–0.59)	<0.0001
LC-Pain in other parts	0.45 (0.32–0.63)	<0.0001

CI, confidence interval; GHS, global health status; HR, hazard ratio; QoL, quality of life; LC, lung cancer; OS, overall survival; PRO, patient-reported outcome.

**Table 6:** OS HRs for a 10-point improvement in PROs from baseline: Time-dependent analyses (*n* = 595).

Scale Name	HR (95% CI)	*p*-Value
GHS/QoL	0.69 (0.64–0.75)	<0.0001
Physical functioning	0.74 (0.69–0.80)	<0.0001
Role functioning	0.78 (0.74–0.82)	<0.0001
Fatigue	0.74 (0.69–0.79)	<0.0001
Pain	0.80 (0.75–0.85)	<0.0001
Dyspnea	0.86 (0.82–0.91)	<0.0001
Appetite loss	0.83 (0.79–0.88)	<0.0001
LC-Dyspnea	0.83 (0.77–0.88)	<0.0001
LC-Coughing	0.84 (0.79–0.89)	<0.0001
LC-Pain in arm or shoulder	0.91 (0.86–0.97)	<0.0001
LC-Pain in chest	0.83 (0.78–0.89)	<0.0001
LC-Pain in other parts	0.90 (0.85–0.95)	<0.0001

CI, confidence interval; GHS, global health status; HR, hazard ratio; QoL, quality of life; LC, lung cancer; OS, overall survival; PRO, patient-reported outcome.

## Data Availability

Qualified researchers may request access to study documents (including the clinical study report, study protocol with any amendments, blank case report form and statistical analysis plan) that support the methods and findings reported in this article. Individual anonymized participant data will be considered for sharing once the product and indication has been approved by major health authorities (e.g., US Food and Drug Administration, European Medicines Agency, Pharmaceuticals and Medical Devices Agency, etc.), if there is legal authority to share the data and there is not a reasonable likelihood of participant reidentification. Requests to access the datasets should be directed to https://vivli.org/ (accessed on 4 March 2026).

## Supplementary Materials

The following supporting information can be downloaded at: https://www.mdpi.com/article/10.3390/cancers18060916/s1, Figure S1: Countries participating in the EMPOWER-Lung 1 and EMPOWER-Lung 3 trials. Figure S2: Kaplan–Meier plots of OS by stable/improved vs. worsened/unobserved PROs in GHS/QoL at (A) 6-, (B) 9-, and (C) 12-month landmark analyses. Table S1: OS HRs of stable or improved PROs vs. worsened or unobserved PROs: Landmark analysis at 6 months (*n* = 444). Table S2: OS HRs of stable or improved PROs vs. worsened or unobserved PROs: Landmark analysis at 9 months (*n* = 321). Table S3: OS HRs of stable or improved PROs vs. worsened or unobserved PROs: Landmark analysis at 12 months (*n* = 251).

## Abbreviations

The following abbreviations are used in this manuscript:

CI	Confidence interval
EORTC	European Organisation for. Research and Treatment of Cancer
GHS	Global health status
HR	Hazard ratio
NSCLC	Non-small-cell lung cancer
OS	Overall survival
PD-1	Programmed cell death-1
PD-L1	Programmed cell death-ligand 1
PRO	Patient-reported-outcome
QLQ-C30	Quality of Life Questionnaire Core 30
QLQ-LC13	Quality of Life Questionnaire Lung Cancer 13
QoL	Quality of life
