# Supplementary material for: Association Between Patient-Reported Outcomes and Overall Survival in Patients with Advanced NSCLC Treated with First-Line Cemiplimab-Based Therapy
Source: Cancers (Basel). 2026 Mar 12;18(6):916. doi: 10.3390/cancers18060916 (PMC13024962; doi:10.3390/cancers18060916)
Supplement: Supplementary file 1 [file cancers-18-00916-s001.zip › cancers-4138453-supplementary.pdf]

Supplementary Figure S1. Countries participating in the EMPOWER-Lung 1 and EMPOWER-Lung 3 trials.

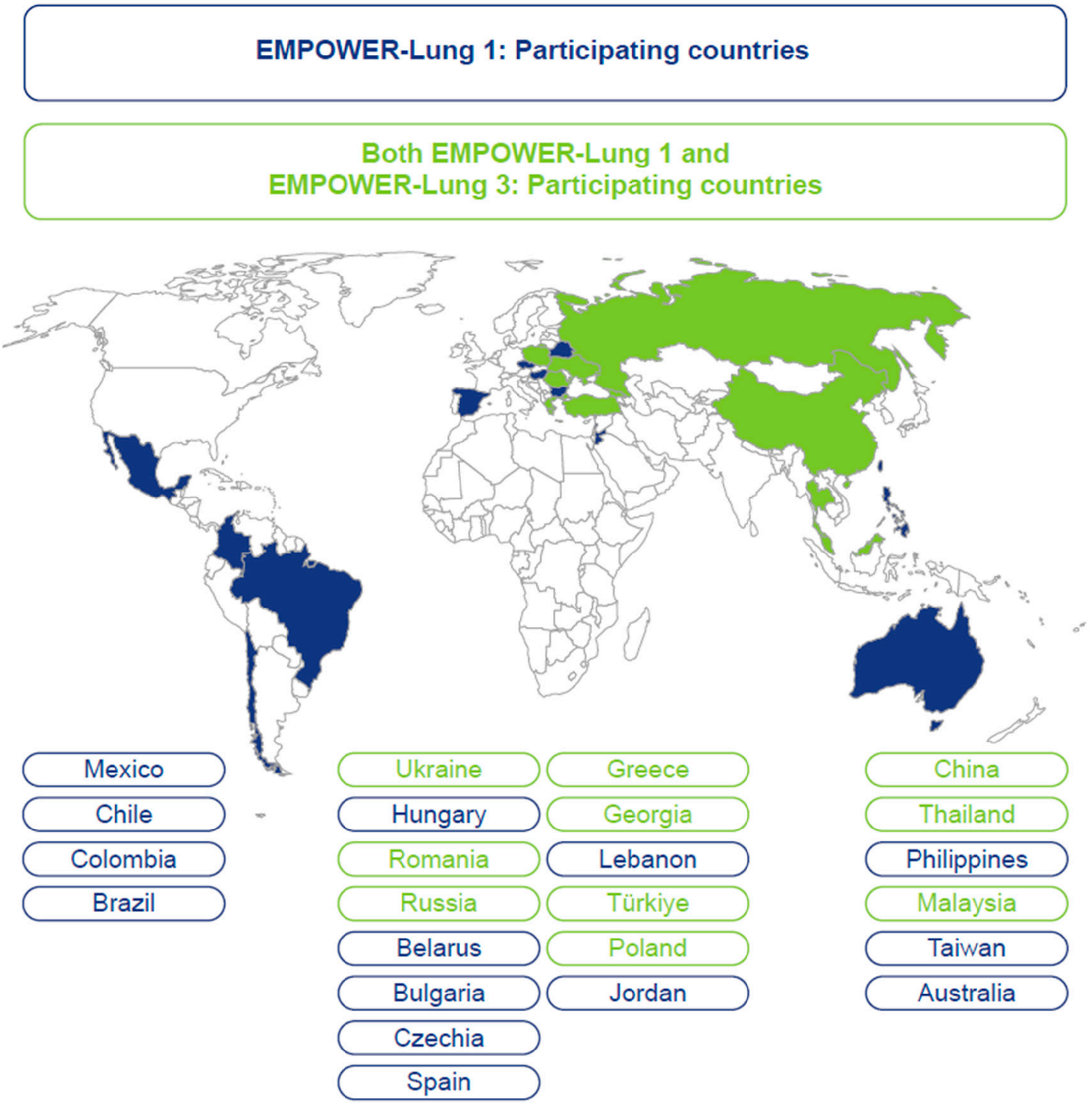

Supplementary Figure S2. Kaplan–Meier plots of OS by stable/improved vs. worsened/unobserved PROs in GHS/QoL at (A) 6-, (B) 9-, and (C) 12-month landmark analyses.

A

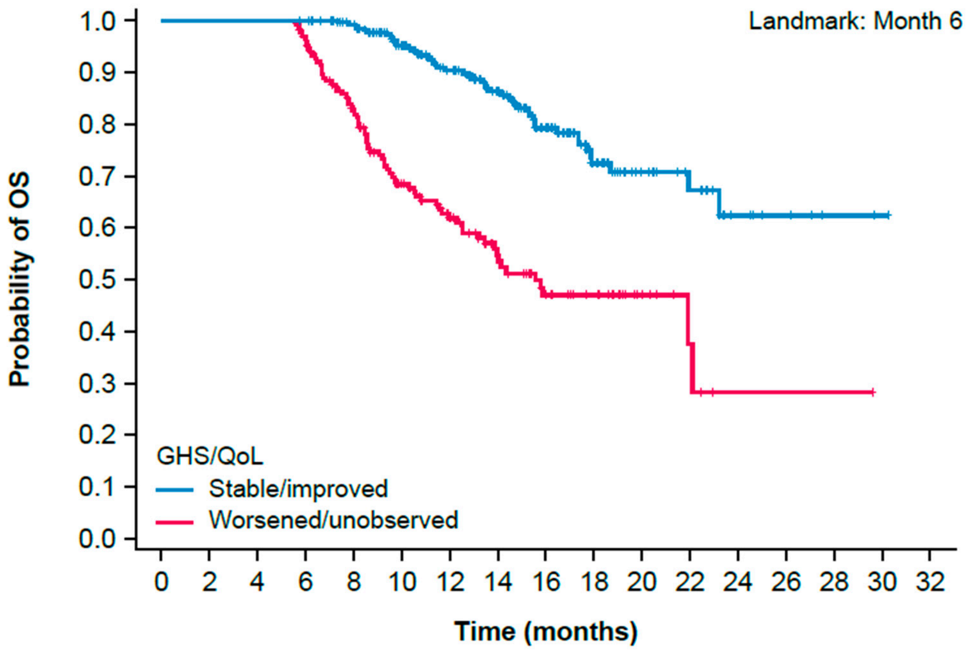

Number of patients at risk

**Stable/improved**	276	276	276	275	260	215	177	139	95	55	27	19	9	5	2	1	0
**Worsened/unobserved**	168	168	168	158	127	92	68	47	33	21	9	4	1	1	1	0	0

B

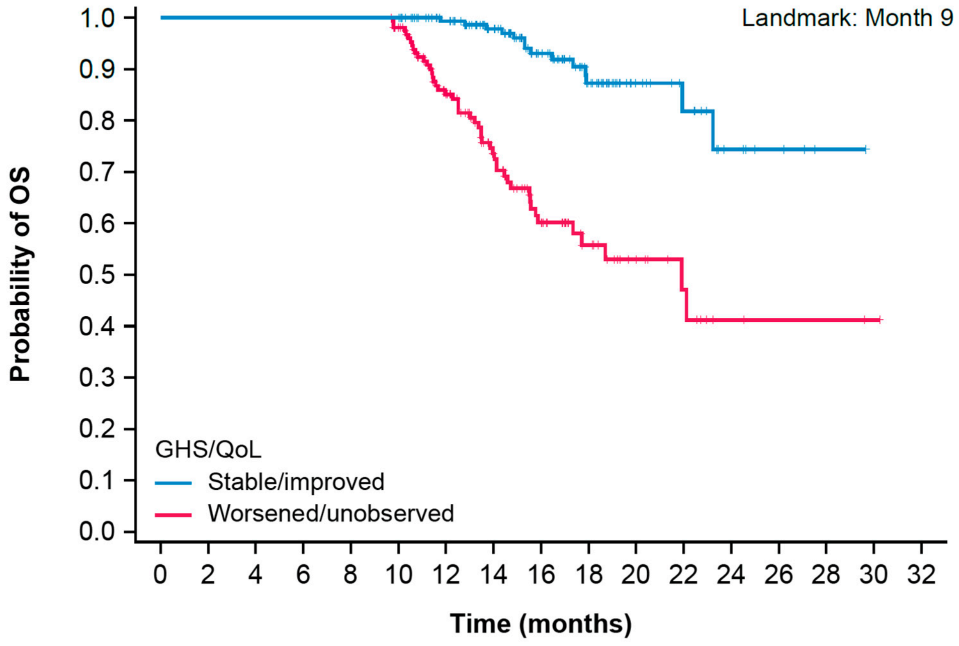

Number of patients at risk

**Stable/improved**	163	163	163	163	163	163	146	119	83	52	23	15	7	4	1	0	0
**Worsened/unobserved**	158	158	158	158	158	144	99	67	45	24	13	8	3	2	2	1	0

C

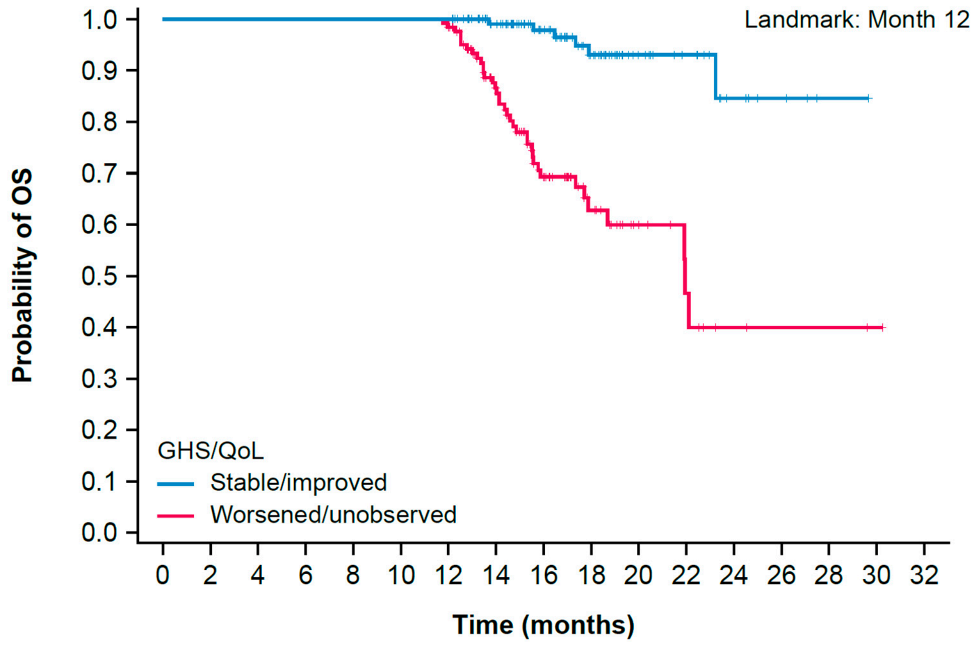

Number of patients at risk

**Stable/improved**	123	123	123	123	123	123	123	103	75	50	24	16	7	4	1	0	0
**Worsened/unobserved**	128	128	128	128	128	128	122	83	53	26	12	7	3	2	2	1	0

GHS, global health status; QoL, quality of life; OS, overall survival; PRO, patient-reported outcome.

Supplementary Table S1. OS HRs of stable or improved PROs vs. worsened or unobserved PROs: Landmark analysis at 6 months (n=444).

Variable	HR (95% CI)	p-value
GHS/QoL	0.29 (0.20–0.42)	<.0001
Physical Functioning	0.26 (0.18–0.38)	<.0001
Role Functioning	0.31 (0.21–0.45)	<.0001
Fatigue	0.41 (0.28–0.60)	<.0001
Pain	0.23 (0.16–0.35)	<.0001
Dyspnoea	0.25 (0.17–0.37)	<.0001
Appetite loss	0.21 (0.14–0.31)	<.0001
LC - Dyspnoea	0.41 (0.28–0.60)	<.0001
LC - Coughing	0.25 (0.17–0.36)	<.0001
LC - Pain in arm or shoulder	0.33 (0.23–0.48)	<.0001
LC - Pain in chest	0.20 (0.14–0.29)	<.0001
LC - Pain in other parts	0.29 (0.20–0.43)	<.0001

GHS, global health status; QoL, quality of life; OS, overall survival; PRO, patient-reported outcome.

Supplementary Table S2. OS HRs of stable or improved PROs vs. worsened or unobserved PROs: Landmark analysis at 9 months (n=321).

Variable	HR (95% CI)	p-value
GHS/QoL	0.14 (0.07–0.27)	<.0001
Physical Functioning	0.15 (0.08–0.29)	<.0001
Role Functioning	0.13 (0.07–0.27)	<.0001
Fatigue	0.21 (0.11–0.41)	<.0001
Pain	0.17 (0.09–0.32)	<.0001
Dyspnoea	0.14 (0.07–0.26)	<.0001
Appetite loss	0.15 (0.08–0.29)	<.0001
LC - Dyspnoea	0.20 (0.11–0.39)	<.0001
LC - Coughing	0.13 (0.07–0.24)	<.0001
LC - Pain in arm or shoulder	0.19 (0.10–0.34)	<.0001
LC - Pain in chest	0.14 (0.07–0.26)	<.0001
LC - Pain in other parts	0.14 (0.08–0.27)	<.0001

GHS, global health status; QoL, quality of life; OS, overall survival; PRO, patient-reported outcome.

Supplementary Table S3. OS HRs of stable or improved PROs vs. worsened or unobserved PROs: Landmark analysis at 12 months (n=251).

Variable	HR (95% CI)	p-value
GHS/QoL	0.10 (0.04–0.26)	<.0001
Physical Functioning	0.16 (0.07–0.36)	<.0001
Role Functioning	0.15 (0.06–0.36)	<.0001
Fatigue	0.12 (0.05–0.32)	<.0001
Pain	0.12 (0.05–0.28)	<.0001
Dyspnoea	0.14 (0.06–0.32)	<.0001
Appetite loss	0.12 (0.05–0.28)	<.0001
LC - Dyspnoea	0.16 (0.07–0.38)	<.0001
LC - Coughing	0.12 (0.05–0.29)	<.0001
LC - Pain in arm or shoulder	0.15 (0.06–0.36)	<.0001
LC - Pain in chest	0.13 (0.06–0.29)	<.0001
LC - Pain in other parts	0.14 (0.06–0.32)	<.0001

GHS, global health status; QoL, quality of life; OS, overall survival; PRO, patient-reported outcome.
